# Working alliance in low-intensity internet-based cognitive behavioral therapy for depression in primary care in Spain: A qualitative study

**DOI:** 10.3389/fpsyg.2023.1024966

**Published:** 2023-03-29

**Authors:** Alberto Barceló-Soler, Javier García-Campayo, Ricardo Araya, Asmae Doukani, Margalida Gili, Azucena García-Palacios, Fermín Mayoral, Jesus Montero-Marin

**Affiliations:** ^1^Navarra Medical Research Institute (IdiSNA), Pamplona, Spain; ^2^Institute of Health Research of Aragon (IIS Aragon), Zaragoza, Spain; ^3^Department of Psychiatry, University of Zaragoza, Zaragoza, Spain; ^4^King’s College London, London, United Kingdom; ^5^Department of Population Health, London School of Hygiene and Tropical Medicine, London, United Kingdom; ^6^Department of Psychology, University of the Balearic Islands, Palma, Spain; ^7^Department of Basic and Clinical Psychology and Psychobiology, Universitat Jaume I, Castelló de la Plana, Spain; ^8^CIBER Fisiopatología Obesidad y Nutrición (CIBERObn), Instituto Salud Carlos III, Madrid, Spain; ^9^Regional University Hospital of Malaga, Malaga, Spain; ^10^Department of Psychiatry, Warneford Hospital, University of Oxford, Oxford, United Kingdom; ^11^Teaching, Research and Innovation Unit, Parc Sanitari Sant Joan de Déu, Sant Boi de Llobregat, Spain; ^12^Consortium for Biomedical Research in Epidemiology and Public Health (CIBER Epidemiology and Public Health—CIBERESP), Madrid, Spain

**Keywords:** working alliance, telemedicine, CBT, qualitative, depression

## Abstract

**Introduction:**

Psychotherapies delivered *via* the Internet have been promoted as an alternative for improving access to psychological treatments. A conceptual working alliance model of blended (i.e., traditional face-to-face consultation combined with Internet-delivered psychotherapy) cognitive-behavioral therapy (b-CBT) for depression has been developed in the UK. However, little is known about how this important therapeutic process, namely the working alliance (WA), is developed and maintained in Internet-delivered cognitive-behavioral therapy without face-to-face consultation (i-CBT). The aim of this study was to evaluate the validity of the WA model of b-CBT in Spanish patients with depression receiving i-CBT.

**Methods:**

Forty-one patients suffering from mild-moderate depression were interviewed to assess their experiences of an i-CBT program. Interviews were conducted with participants who received a self-guided application (*n* = 9), and low-intensity support (*n* = 10). Three group interviews were also conducted with patients who either did not start the program (*n* = 8) or did not complete it (*n* = 6), and with patients who did complete it (*n* = 8).

**Results:**

Qualitative thematic content analysis was performed using the constant comparative method, which revealed four main themes: “bond,” “goals,” “task,” and “usability heuristics,” all consistent with the existing literature. However, a new subcategory emerged, called “anonymity,” which may highlight the social stigma that mental illness still has in the Spanish context.

**Conclusion:**

Results suggest that the development and maintenance of the WA through i-CBT could offer a better experience of the therapeutic process and improve the clinical impact.

**Clinical Trial Registration:**

Clinicaltrials.gov, identifier: NCT01611818.

## 1. Introduction

Depression is the world’s most prevalent 12-month mental health condition, affecting nearly 300 million people (World Health Organization, 2021), including 44 million in Europe and 2.4 million in Spain. It was also the third leading cause of years lived with disabilities in 2017 (World Health Organization, 2008). Several epidemiological studies have concluded that depression has a great impact on health due to its negative impact on the professional development of people as it is related to job loss and incapacity for work, as well as intra- and extra-family social relationships (Alonso et al., 2004; Kessler and Bromet, 2013; McKeever et al., 2017). Furthermore, depression rates appear to have worsened in the general population as a result of the recent COVID-19 pandemic (McKeever et al., 2017; Bueno-Notivol et al., 2020; Planchuelo-Gómez et al., 2020), which also resulted in restricted access to mental health services during the different lockdowns (Talevi et al., 2020). Over the years, the scientific community and mental health professionals have been advocating the importance of implementing innovations from research into mental health care in order to improve strategies to address depression. This would make it possible to overcome the difficult access to mental health services caused by long waiting lists in public health systems, and the high economic cost of private care. This is a significant problem because it means that a large part of the population does not obtain the necessary treatment (Andrade et al., 2014; Tristiana et al., 2018). There is evidence that psychological therapies delivered *via* information and communication technologies (ICTs), such as Internet-delivered cognitive behavioral therapy (i-CBT) programs, could contribute to improving access to mental health care (Turner et al., 2013; Andrade et al., 2014; Li et al., 2015; Tristiana et al., 2018; Gillard et al., 2021).

Available meta-analyses and systematic reviews on the efficacy of Internet- and computer-based psychotherapies indicate that they are effective in reducing depressive symptoms, with standardized effect sizes ranging from 0.27 to 0.90 (Richards and Richardson, 2012; Josephine et al., 2017; Karyotaki et al., 2017; Rogers et al., 2017). Assistance or support from a health professional was found to improve outcomes, with standardized effect sizes varying between 0.47 and 1.00 (SPEK et al., 2007; Richards and Richardson, 2012; Cowpertwait and Clarke, 2013; Massoudi et al., 2019). However, while the findings on treatment effectiveness of i-CBT is encouraging, psychological interventions applied *via* ICTs continue to present certain problems, including a high drop-out rate (Edmonds et al., 2018; Schmidt et al., 2019). This may be enhanced by such limitations as lack of esthetic appeal, usability problems, generic programs that cannot be tailored to patient needs, and security and confidentiality concerns, among others, that may impact users’ experience of the treatment, in addition to important psychotherapeutic processes such as the working alliance (WA; Edmonds et al., 2018).

WA refers to the client-therapist relationship in a therapeutic context. According to Bordin (1979), it is defined as the active collaboration between therapists and patients that involves three fundamental dimensions: (1) setting therapeutic *goals* according to the client’s needs and perceptions, (2) the agreement established regarding the specific *tasks* to be carried out by both parties to achieve those goals, and (3) the *bond* that is created between the patient and the therapist by working together in the same direction. For decades, the impact of the client-therapist relationship on clinical outcomes has been one of the main topics of study in the field of psychotherapy (Horvath, 2000).

Bordin’s conceptualization (Bordin, 1979) was contextualized in the field of traditional face-to-face psychotherapy and may present certain limitations in the new therapeutic contexts brought about by i-CBT interventions (Gómez Penedo et al., 2019). It is therefore necessary to attempt to understand the relevance of WA in i-CBT. A conceptual WA model for a blended (i.e., traditional face-to-face consultation combined with Internet-delivered psychotherapy) cognitive behavioral therapy (b-CBT) for depression has recently been developed in the United Kingdom (Doukani et al., 2020). This model adds a fourth element to Bordin’s conceptualization, which is categorized as *usability heuristics* and refers to the use of digital technologies to promote active engagement, self-discovery, and autonomous problem-solving in b-CBT (Doukani et al., 2020). This highlights how the use of technology in psychotherapy can impact patients’ WA needs. It should also be noted that there were some nuances in relation to how clients experience Bordin’s original *bond*, *task*, and *goals* categories. The details on how these dimensions are defined and structured by key features can be found in Table 1. In the *bond* category, both the mental health professional and the computerized program were identified as playing an important role in patients’ therapeutic experience, although the human factor continued to be a fundamental element. With regard to *goals*, together with the collaborative work between the therapist-patient, the computerized program itself should also be included as a key factor in identifying patients’ expectations. Finally, in the *task* dimension, two specific subsections were identified: first, the “activity-based task” refers to patients’ own ability to perform the tasks; and second, “responsive support” involves the professional’s ability to adequately respond to a variety of expressed and unexpressed patient needs. The authors concluded that patients considered both therapists and the program as important in order to meet their WA needs (Doukani et al., 2020).

**Table 1:** Theme/subtheme definitions of the WA for b-CBT analytical framework (Doukani et al., 2020).

Themes/subthemes	Conceptual definitions
BOND	*[Competencies of the mental health professional that allow the establishment/maintenance of a working relationship with a patient].*
- Feeling understood	The mental health professional transmits active listening and demonstrates empathic awareness and knowledge of what the patient is concerned about.
- Genuineness	Involvement in the patient’s therapeutic process that is perceived as authentic.
- Partnership	Friendship/teamwork between the patient and the mental health professional.
GOALS	Identification of what the patient wants to achieve that arises from the collaborative work with the mental health professional.
TASKS	[*Set of activities agreed between the patient and the mental health* professional *to achieve the set goals*].
Activity-based task	[*Patient’s ability to work on tasks*].
- Personalized	Ability to adapt the agreed therapeutic process to the patient’s needs.
- Useful	Promotion of new learning, reflection, and desired change in the patient’s life.
- Complementary	Continuity derived from face-to-face sessions and those of the computerized program, in such a way that they are considered coherent.
Responsive support	[*Skills of the mental health* professional *to respond to the patient’s needs, with a therapeutic orientation that creates a safe space where the patient feels comfortable*].
- Accountability	Mental health professional as a figure that generates commitment to the intervention.
- Guidance	Provision of guidance and reassurance on therapeutic tasks.
- Expression of feelings	Interaction that facilitates expression of issues that are relevant to the treatment process.
USABILITY HEURISTICS	*[Use of technology to promote engagement, self-discovery, and autonomous problem-solving].*
- Accessibility	Facilities to access to the online psychotherapy program at a time and place of convenience.
- Interactivity	Characteristic of computerized programs consisting in reacting to customer responses and responding accordingly.
- Ease of use	Way with which the online psychotherapy program is handled through the interface.
- Esthetic appeal	Appearance or attractiveness of the online psychotherapy program interface.
- Self-directed	Patient’s ability to assume responsibility to direct their own therapeutic process, generating independence and control.

Moving forward, it is important to consider the nature of the intervention being applied, given that psychotherapeutic designs delivered *via* ICTs can vary by the intensity of the contact established between the professional and the patient. There are programs that are completely guided by a supervisor (guided), as well as interventions that are fully self-taught by the patient (unguided; Karyotaki et al., 2021; Köhnen et al., 2021). Despite the increased number of studies exploring treatment outcomes of i-CBT, there is little research that conceptually delves into the nonspecific therapeutic mechanisms that influence clinical improvement, such as the WA, in relation to different Internet/computer-based interventions and according to the degree of client-therapist contact in which blended programs constitute the highest level of professional support, followed by guided programs (consisting of little support) and unguided programs that offer no professional support (Zagorscak et al., 2020).

The aim of this study was to evaluate the validity of the WA model for b-CBT (Doukani et al., 2020), in the context of an i-CBT intervention with patients suffering from mild to moderate depression –and thus, patients who receive less professional support compared to those from the original study conducted in the United Kingdom. For this purpose, we used one group with guided i-CBT and another group without any professional support by means of unguided i-CBT in the Spanish primary care (PC) setting.

## 2. Methods

### 2.1. Design

A qualitative design was used to gain an in-depth understanding of participants’ experiences of WA in two low-intensity i-CBT conditions, guided and unguided, for depression in the PC setting in Spain. We applied Malterud’s guidelines for conducting and reporting qualitative research (Malterud, 2001).

### 2.2. Data collection

Participants taking part in a randomized controlled trial (RCT) for the purpose of comparing the effectiveness of a low-intensity therapist-guided i-CBT or a completely self-guided i-CBT, with treatment as usual-care for depression, were invited to take part in a qualitative study. Participants in the trial were recruited from PC community health centers during routine clinical practice in three regions of Spain (Aragon, Andalusia, and the Balearic Islands). This project was part of a mixed-method research approach that included a multicenter RCT with depressive patients. It was approved by the Research Ethics Committee of the regional health authority of the Autonomous Community of Aragon, Spain (ref: PI10/039), which covered all the other participating autonomous communities (López-del-Hoyo et al., 2020).

PC professionals identified patients who could benefit from the intervention during routine clinical practice at PC centers. For example, doctors who detected a patient with symptoms of depression in their offices could proceed to explain to them the research study and its characteristics if they considered it pertinent. People who were interested in taking part in the study were required to meet the inclusion criteria, which included meeting the ICD-10 diagnostic criteria for Major Depressive Disorder, being diagnosed with the MINI Neuropsychiatric Interview in Spanish (Lecrubier et al., 1997; Ferrando et al., 1998), and scoring 14–28 points on the Spanish version of the Beck Depression Inventory-II (BDI-II), which corresponds to a mild–moderate severity cut-off point (Organización Mundial de la Salud, 1992; Sanz et al., 2005). In addition, the BDI-II scores of study participants at 12 months follow up were also monitored. However, people were excluded from the study if they: (a) were aged under 18 years or over 65 years, (b) had written/spoken language difficulties that would prevent them engaging with the computer-assisted psychotherapy program, (c) had comorbid DSM-IV Axis I mental disorders (e.g., psychotic disorders, dementia), and (d) those who manifested low affinity to the use of technologies (by means of the following question: “what is your level of affinity for new technologies?” with “high,” “intermediate,” and “low” as possible responses). All participants had to sign a written informed consent form in order to be included in the study.

Patients were intentionally selected (e.g., in terms of age, gender, residential environment, educational level, and clinical improvement) to participate in individual or group interviews, while maintaining the necessary homogeneity, but also potential discursive heterogeneity, of the original study sample. Discursive heterogeneity was also linked to different levels of participation (e.g., starting treatment vs. not starting treatment, and dropping out of treatment vs. completing treatment) and receiving different treatment formats (i.e., low-intensity support or self-guided application; López-del-Hoyo et al., 2020). Individual interviews were conducted with patients from both the low-intensity support group (*n* = 10) and the self-guided group (*n* = 9). Three group interviews, with patients from the two intervention groups, were conducted representing the three levels of completion outlined, thus consisting of patients who: (a) did not start the program (*n* = 8), (b) started but dropped out of the program (*n* = 6), and (c) completed the intervention as intended (*n* = 8). No patients refused to participate in the individual interviews. However, some of them did not attend the group interviews, each of which originally included 12 participants. Considering potential absences, we expected that between 6 and 12 participants would finally participate in each group and, therefore, produce enough interactions so as to be productive in terms of discourse. The total number of participants was finally considered adequate, as data saturation was obtained (Phillips and Hardy, 2002).

These two qualitative research approaches (i.e., individual and group interviews) were proposed to complement each other, with the individual interviews exploring the topic list to generate tentative hypotheses, and the group interviews favoring greater productivity of results and detail in terms of the different discursive positions. The participant sociodemographic characteristics are presented in Table 2. Most participants were aged between 41 and 60 years (69%), female (78%), and lived in an urban area (90%). Most had also completed university studies (41%) and experienced low clinical improvement (51%) after receiving the corresponding i-CBT intervention.

**Table 2:** Characteristics of participants (*n* = 41).

Stratification variables	Interviews		Groups			
	Self-guided application	Low-intensity support	Not start	Drop out	Completed successfully	Total
	*n* = 9	*n* = 10	*n* = 8	*n* = 6	*n* = 8	*n* = 41
**Age**						
20–40 years	3 (7%)	3 (7%)	2 (5%)	3 (7%)	1 (2%)	12 (29%)
41–60 years	6 (14%)	7 (17%)	5 (12%)	3 (7%)	7 (17%)	28 (69%)
>60 years	0 (0%)	0 (0%)	1 (2%)	0 (0%)	0 (0%)	1 (2%)
**Sex**						
Male	2 (5%)	2 (5%)	1 (2%)	2 (5%)	2 (5%)	9 (22%)
Female	7 (17%)	8 (20%)	7 (17%)	4 (10%)	6 (14%)	32 (78%)
**Residential setting**						
Urban	9 (22%)	9 (22%)	8 (20%)	6 (14%)	5 (12%)	37 (90%)
Rural	0 (0%)	1 (2%)	0 (0%)	0 (0%)	3 (7%)	4 (10%)
**Level of education**						
Primary	3 (7%)	3 (7%)	3 (7%)	3 (7%)	1 (2%)	13 (32%)
Secondary	2 (5%)	2 (5%)	3 (7%)	3 (7%)	1 (2%)	11 (27%)
University	4 (10%)	5 (12%)	2 (5%)	0 (0%)	6 (14%)	17 (41%)
**Clinical improvement**						
High	4 (10%)	5 (12%)	0 (0%)	0 (0%)	4 (10%)	13 (32%)
Intermediate	2 (5%)	3 (7%)	0 (0%)	0 (0%)	2 (5%)	7 (17%)
Low	3 (7%)	2 (5%)	8 (20%)	6 (14%)	2 (5%)	21 (51%)

SGA: self-guided application group. LIS: low-intensity support group.

Both individual and group interviews were initially guided by the topic list (Table 3), although the group interviews also built on the results of the individual interviews so as to gain a better understanding by taking advantage of participants’ interactions in the groups. Direct references to WA were not made in order not to determine the participants’ discourse with the theoretical assumptions of the analytical framework used (Doukani et al., 2020), focusing instead on general issues related to engagement (Table 3). This allowed us to achieve standardization in the interview sessions in an open and flexible way, while also including issues introduced by the participants (Brown, 1999). The initial topics to be explored were selected by an interdisciplinary panel of experts in the delivery of mental health interventions *via* ICTs (two clinical psychologists, one psychiatrist, and one sociologist), and included key issues that had been identified in previous research, such as training in new technologies and opinions regarding the use of mental health interventions *via* ICTs (Waller and Gilbody, 2009), information needed to cope with the online therapeutic process (Barney et al., 2011), personal preferences, program content, identification with the program, material conditions for practice, symptomatic change experiences, professional support and adherence (Gerhards et al., 2011), accommodation of therapy into the daily routines, development of a virtual/anonymous relationship with the therapist, online communication of thoughts and emotions, the possibility of revising written materials generated through the therapeutic process (Beattie et al., 2009), facilitators/barriers, expectations, attitudes, and continual engagement with the program (Bendelin et al., 2011), From the list, inquiry processes were directed toward understanding how the intervention *via* ICTs might modify the therapist-patient relationship. Further details of the qualitative study protocol can be found elsewhere (Montero-Marín et al., 2013).

**Table 3:** Topic list.

Areas	Issues
Computer aspects	- Information technology skills
	- Material resources
	- Routine use
Expectations	- Therapeutic expectations
	- Online vs. face-to-face therapy
Experiences	- Identification with the program
	- Barriers and facilitators
	- Changes in symptoms
Attitudes	- Expressed possibilities
	- Anonymous and virtual relationship
	- Reflectivity of responses
Information	- Information and preferences
Staff	- Normalization in primary care
	- Professional support
Improvements	- Interface
	- Program
	- Adherence

Individual in-depth interviews were conducted by two interviewers in an indirect and nondirective way, with the study topics introduced openly and progressively. In view of the content analysis adopted by the study, nonverbal responses were noted for consideration alongside the audio recordings. The group interviews were moderated by two researchers: the main interviewer and an observer. The role of the main interviewer consisted of gradually and indirectly introducing the topics, promoting dialog, and steering productive participation. The observer’s role consisted of welcoming participants and taking field notes to provide additional information to the verbal data obtained, such as information regarding nonverbal language, responses to the moderator’s interventions, and contextual aspects. Both individual and group interviews were conducted/supervised by researchers who had no prior contact with the patients, avoiding any previous relationship that could affect how the interviews unfolded and influence the quality of responses obtained, thus reducing the risk of desirability bias. The interviewers were two female clinical psychologists who were familiar with the WA concept and with previous experience in conducting interviews and qualitative research.

The interviews were conducted in neutral rooms allocated to the research team and set up for this purpose at the tertiary referral hospital of each PC center in the cities involved in the study. Only patients and researchers were present. The individual and group interviews lasted between 60 and 90 min. Audio recordings were made of their contents and a verbatim transcription subsequently produced.

### 2.3. Intervention

The intervention was “Smiling is Fun,” an Internet-delivered program based on CBT (i.e., i-CBT) that comprised 10 modules and had a duration of 12 weeks (Montero-Marín et al., 2016). The program was configured to send a confirmation email to the patient upon completion of each module on the website. Furthermore, in both treatment conditions (i.e., low-intensity support and self-guided application), the program would send an automatic email to both the patient and the therapist when more than 2 weeks had passed without the user logging into the program. This message was intended to encourage the patient to continue working to benefit from the intervention. In guided i-CBT, a psychotherapist contacted the patients to offer them help (e.g., to ask about any difficulties or problems in complying with the program), and facilitate completion of the intervention within the timeline. The patient could contact the psychotherapist by phone to ask questions or seek advice for a maximum of 1 h during the treatment period. In contrast, unguided i-CBT did not offer contact with any psychotherapist during the treatment period. Both groups could obtain technical assistance from a technician *via* email regarding operation of the program (López-del-Hoyo et al., 2020).

### 2.4. Data analysis

Initially, a thematic content analysis (Braun and Clarke, 2006) of the individual interviews was performed in order to contrast the theoretical WA model for b-CBT developed in the United Kingdom (Doukani et al., 2020). Moreover, grounded theory, which allows models to be developed from qualitative data (Corbin and Strauss, 2008), was also used to identify possible new categories that reflected potentially idiosyncratic properties. In addition, analysis of the individual interviews was complemented by analysis of the group interviews by means of the sociological model, which involved the identification of the explanatory axes according to participants’ different discursive positions in terms of the level of guidance received (i.e., low-intensity support or self-guided application), as well as completion status (i.e., not starting treatment, dropping out of treatment, or completing treatment; Alonso, 1998; Phillips and Hardy, 2002). Data from the different groups were analyzed separately (intra-group analysis), and then were regrouped to compare the relevant topics (inter-group analysis).

These analytical procedures were independently carried out by two researchers, clinical psychologists who were familiar with the concept of the WA and with experience in qualitative data analysis. They were not involved in conducting either the individual or group interviews. Discrepancies between the two analysts were solved by consensus. MAXQDA-2020 (VERBI Software, 2019) qualitative data analysis software was used. The analysis was iteratively performed by the analysts (*n* = 2), who were not directly involved in the data acquisition but proposed tentative hypotheses; and the researchers (*n* = 2), who conducted the individual/group interviews and refined the search for information from the initial topic list toward the evaluation of tentative hypotheses; and finally participants (*n* = 5), who provided their agreement with the interpretations once data saturation was reached (Phillips and Hardy, 2002; participants provided their agreement with the interpretations by reading and endorsing a report that included the results section). The triangulation of procedures, analysts, and techniques allowed increasing consistency and rigor, maximizing the breadth and depth of interpretations.

## 3. Results

**Figure 1:**
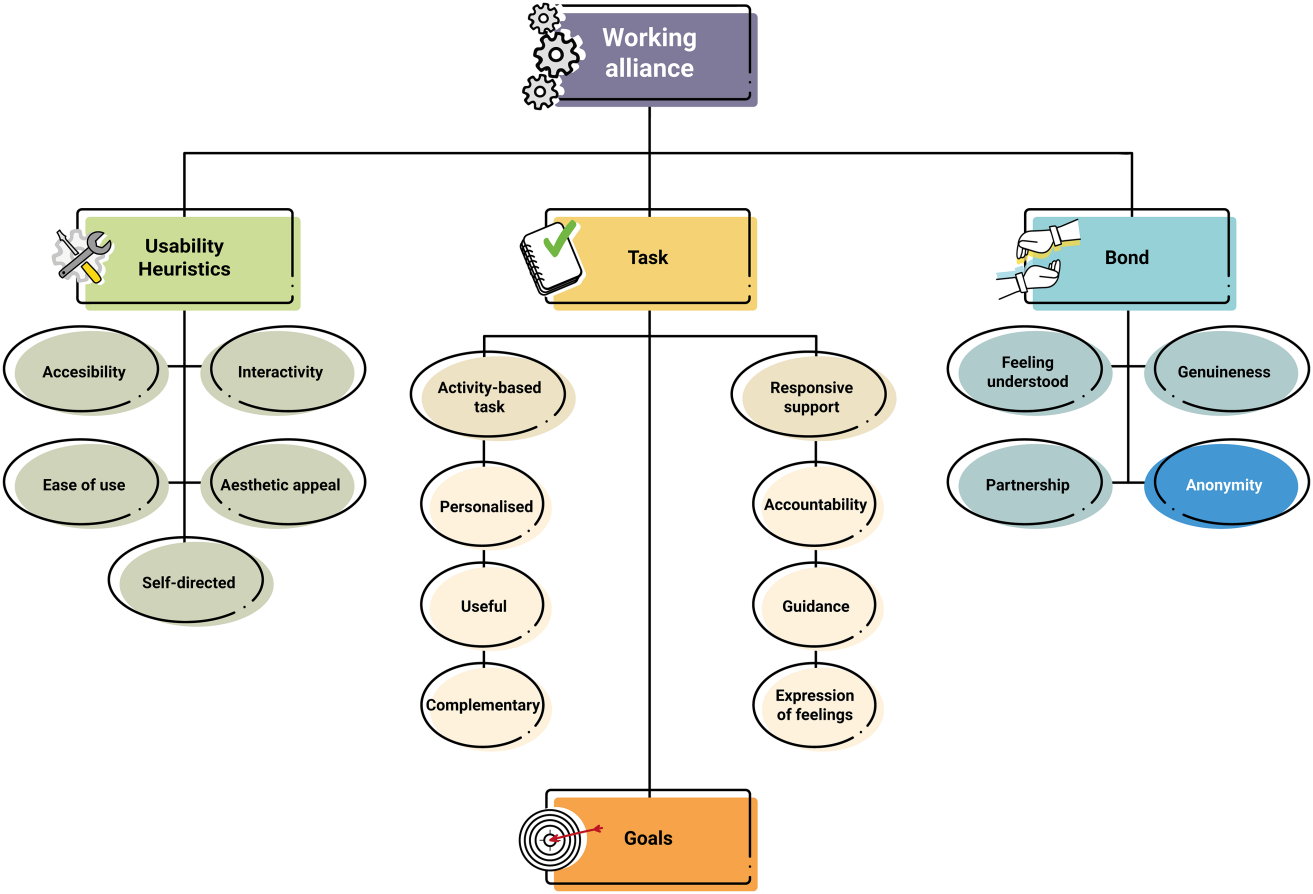
Working alliance demands in a low-intensity online CBT intervention. The new category added to the model that emerged in the Spanish Primary Care setting in patients with mild–moderate depression (i.e., “Anonymity”) is highlighted. Figure adapted from Doukani et al. (2020).

The results obtained from the data analysis showed a good fit of the WA theoretical model for b-CBT (Doukani et al., 2020) in the context of PC in Spain using i-CBT, with the same main themes: “bond,” “goals,” “task,” and “usability heuristics.” However, a new subcategory clearly emerged in the present study (Figure 1), called “anonymity,” which was added to the model under the “bond” category. Moreover, new nuances also emerged in the “usability heuristics” category. These were integrated into the existing subcategories of “accessibility” and “ease of use,” increasing the model’s original conceptual density.

### 3.1. Bond

The “bond” dimension was positioned as the main component in the development of an adequate initial interaction with the therapeutic program by the patient. In this sense, participants appeared to underscore the superiority of the human factor over the digital program with respect to “feeling understood”:

*“[Describing my emotional state] every day or almost every day, whenever I logged in, how I was feeling… Well, that was really hard; while if you tell it to a person, it’s like they understand you, like… like they empathize with you; but with a computer, you know that nope, there’s nothing… You type your answer in, and that’s it; there’s nothing else, it’s only purpose is to be a tool that lets you see if you’re getting better or worse, but without giving you any feedback.”* [Woman, 44 years old, low-intensity support group, dropped out due to worsening symptoms, BDI score not available (BDI scores are unavailable for patients who did not complete all their follow up assessments)].

The concept of “genuineness” was related to the perceived efforts and involvement of professionals in the creation of an adequate WA. Participants suggested that it was a key element that influenced the process of establishing a relationship with the therapist, and that held important implications for actual clinical improvement:

*“… This way you’re finding out things that otherwise would never come out. I mean, he can ask you, ‘What is there? What can you see here?’ And I tell him, ‘Well, I don't see anything.’ And he tells you, ‘Well, you will see something.’ [And I say,] ‘No, no, I don't see anything. Let’s see, you’re asking me this because it’s a test, or because of this or that, and… and my answer is, Nothing!’ And it's there, and even if you ask me again, well, eh, in person. Well, this man, he turns it around and comes to you from a different angle and asks you again. And in the end, it turns out that where you didn't see anything, you say, ‘My God! well, yeah… there was something!’ He helps you to find that … that something.”* [Man, 47 years old, low-intensity support group, BDI score: 13 (12-month follow up)].

The “partnership” theme refers to the establishment of a working relationship comparable to a friendship between the patient and the therapist and/or computer program. Participants stated that both warmth and perceived trust about the program were important, regardless of receiving support from a professional or not:

*"I thought it showed quite a lot of warmth for a computer program… But, of course, that depends on the person [referring to the individual characteristics of each person who uses the program], on each person’s attitude.”* [Woman, 46 years old, low-intensity support group, BDI score: 2 (12-month follow up)].

*“No, I did not find the computer program to be at all suspicious; I trusted it completely.”* [Woman, 56 years old, low-intensity support group, BDI score: 28 (12-month follow up)].

However, one participant in the self-guided group also described a feeling of loneliness and of being “more by yourself.” This might indicate that guidance could be an important aspect for the establishment of a working relationship:

*“Logically, the whole thing about using a computer was always going to be much colder, right? You’re going to feel more… lonely, or more by yourself.”* [Woman, 44 years old, self-guided application group, dropped out due to family problems, BDI score not available].

The last subcategory integrated into the bond theme, and one that emerged as a new topic in the Spanish sample was “anonymity.” This concept was consistently raised by participants. They appeared to value the anonymity of a digital intervention program in light of existing prejudice and stigmatization of mental illness and the use of mental health services by society, and the shame that participants feel because of this:

*“For me, anonymity is an essential part of this… Because I’m actually very ashamed to say that I have depression, so, mmm, because it’s something I don’t talk about, it’s a burden that I carry; I mean, if I would have had to give my name, I wouldn't have done it.”* [Woman, 52 years old, self-guided application group, BDI score: 30 (12-month follow up)].

*“I’ve never had a problem with saying that I go to the psychiatrist, because I think that the head is a part of the body. And that there are professionals for everything; in other words, there’s a professional to treat your head, to treat your leg, for everything, so never a problem… I don’t say it all the time, but whenever I have to say it, I do. But, of course, it’s always convenient, with some issues, to have a bit of anonymity, because people don’t really understand the idea of going to a psychiatrist, because they mistake it for insanity, for people who are in a pretty hopeless situation, with… then in that case, anonymity is good…”* [Woman, 56 years old, low-intensity support group, BDI score: 28 (12-month follow up)].

“Interviewer: So, do you think that the anonymity offered by online therapy is a bonus?

*Patient: Yes, one hundred percent. They don’t know who you are, they don’t know what, what you look like. As far as I’m concerned, totally, one hundred percent.”* [Man, 47 years old, low-intensity support group, BDI score: 14 (12-month follow up)].

### 3.2. Goals

The “goals” theme was also identified as an element of major significance. Participants reported that activities focused on setting goals were important for the maintaining and expanding the initially established therapeutic bond, for motivating participants to achieve symptomatic improvement. Goal-setting was also identified as helpful for promoting greater awareness of the progress made on their treatment journey:

“Interviewer: Which module do you think has been the most useful for you?

*Patient: I think that the one that most… uh… the ones that have been most helpful were the ones where you start… uh… to set your goals; I think it’s super important because it’s the moment you begin to realize where you are and where you want to go, and without doing this, well… you also realize what’s stopping you, what’s been stopping you from doing it, the feelings that have been such a burden for you.”* [Woman, 48 years old, self-guided application group, BDI score: 5 (12-month follow up)].

### 3.3. Task

The third main theme identified was the so-called “task.” This element is divided into the following two subcategories: “activity-based task” and “responsive support,” which were endorsed by their respective subthemes.

#### 3.3.1. Activity-based task

“Activity-based task” comprises the “personalization” of tasks in relation to patients’ needs; the “usefulness” of the tasks in terms of encouraging learning and reflection while motivating patients to change their life; and the “complementary” nature of traditional therapy tasks performed face-to-face and those delivered *via* the Internet, which are experienced by the participant as coherent and continuous.

With regard to “personalization,” patients stressed the importance of feeling motivated to complete a therapeutic program that includes content that is specific to their concerns, as opposed to a generic program. Participants also stated their preference to have access to more material that addressed their specific difficulties as they benefited less from being exposed to a wide variety of topics:

*“There are things that may interest you more than others. There are sections where you say, well… this here, well, it’s not my problem, I might have to skip it. But then you come across a section, a problem, where it can be of help to you. So, there are topics that have nothing to do with you, or maybe only a little… And there are other topics that could really help you, and you tell yourself that a little more information, or reading more, or… more examples wouldn’t be a bad thing… Do you follow me?”* [Man, 47 years old, low-intensity support group, BDI score: 14 (12-month follow up)].

In terms of the “usefulness” of the intervention, participants reported that some aspects of the online program that they regarded positively increased their engagement with the intervention. They included aspects that facilitated openness and mental flexibility, and those providing tools that would allow them to put the theoretical contents into practice, to apply what they had learned:

*“It has been useful to me, it has opened me up, because it changes the way you think and opens your mind; it gives you new ideas, ideas that, you know, that… putting them into practice can be very, very useful, very useful. It opens up your way of thinking; it makes it more flexible, and you say, ‘Let's see, let's see, let's see.’ You stop, and you remember things, that… that you can use and that… the habits you have, the ones that aren’t good or appropriate for you, well, well, and you change them. Yes. In fact, I think it has been a good experience"* [Woman, 46 years old, low-intensity support group, BDI score: 0 (12-month follow up)].

In relation to the “complementary” category, all patients who had access to the digital platform said that, despite perceiving it as a useful and even necessary tool, the digital intervention program could not totally replace the healthcare professional. This was particularly the case for those who identified themselves as suffering from a deep depression that prevented them from starting with the program:

*“I think this type of program is a tool for support, but it could never ever replace a professional.”* [Woman, 46 years old, low-intensity support group, BDI score: 2 (12-month follow up)].

“*It depends on the stage of depression you’re at … If you’re at a stage where you’re able to log into the program, and it helps you … In other words, when what you’re reading helps yourself to improve, then fantastic … we are achieving the goal. Now, if you’re in a very deep depression … maybe … even if you know what you are reading,. you won’t be able to put it into practice in your everyday life*”. [Man, 47 years old, self-guided application group, BDI score not available)].

#### 3.3.2. Responsive support

The second subdivision of “task” is called “responsive support” and includes three subcategories. The first is “accountability,” which refers to the presence of a human facilitator that provides positive feedback to help patients engage with the therapeutic tasks. The second is “guidance,” a reference to the therapist’s availability during the intervention process to provide patients with suggestions that otherwise might not arise without their involvement. The third subcategory is “expression of feelings,” through which participants express their need to share their internal experiences, including the possibility of writing comments to advance in the therapeutic process. However, participants suggested that it was important for a safe space to be created in therapy to facilitate this.

Participants admitted that the presence of a person to supervise their progress on the program and their performance of the tasks set by the different modules produced a greater feeling of “accountability” over the therapeutic process, facilitating commitment to achieve clinical improvement:

*“When it comes to the computer, when you want to do it, you do, but if you haven’t in two or three days, even if you turn on the computer, you might feel a little guilty, and you tell yourself, ‘Oh! It’s already been two days!’ But, if you’re dealing with a person, you can’t do that. It involves a big commitment and you must be there; you can’t let them down. At least that’s how I see it.”* [Woman, 46 years old, low-intensity support group, BDI score: 0 (12-month follow up)].

Similarly, patients indicated that being able to count on a person who could “guide” them through the program and could solve any doubts that might arise gave them peace of mind and security to continue moving forward with a greater sense of control regarding the therapeutic process:

*“Of course, it shows when you don’t have a person by your side to guide you a little or… resolve doubts… It's more about the therapy, not me; when it comes to seeing the psychologist, for example––I’ve never been to one––but I imagine that a psychologist can guide you better … They can guide you more, or they can… more than, more than on a course.”* [Man, 47 years old, low-intensity support group, BDI score: 14 (12-month follow up)].

This aspect is important enough to raise feelings of isolation and entail a risk of the participant dropping out of the program if it is not fulfilled:

*“Well, it would be good if … if there’s a follow-up. If the psychologist or psychiatrist who treats you follows up your case, of course. If the professional is watching the day-to-day process of how you’re getting on, your state of anxiety, your, your situation. Yes, if someone does a follow-up on that… [Doing it online] gives me the impression that… well, that it’s you alone with the machine and that nobody is following up, nobody is concerned, because, in my case, I was getting worse…”* [Woman, 44 years old, low-intensity support group, dropped out due to worsening symptoms, BDI score not available)].

Finally, the data show the value of having a person to whom patients can tell their problems. This is related to patients’ need to be able to “express their feelings” to another person who will listen and understand without judging:

*“Talking to someone helps; maybe it just helps to talk and being able to demonstrate, externalize [feelings, concerns…] and so on. So maybe a machine won't give it to you.”* [Man, 47 years old, low-intensity support group, BDI score: 13 (12-month follow up)].

With regard to this need to express, the program’s relative lack of responsiveness was highlighted, particularly in the self-guided application group:

*“Very limited means of expression because all you had to do was to fill in a few items… it was a grid. I mean, there was nowhere for you to put, mmm… For example, it would have been nice for each module to have a space where you could leave a personal comment…”* [Woman, 52 years old, self-guided application group, BDI score: 30 (12-month follow up)].

*“The answers were a bit limited… Those given by the computer I think were a bit limited because sometimes, hmm … I would have given another answer, but I had to limit myself … I think the program should have left a blank space so that you could have said something else.”* [Woman, 50 years old, self-guided application group, BDI score: 0 (12-month follow up)].

#### 3.3.3. Usability heuristics

The fourth and last main theme of the WA model for b-CBT (Doukani et al., 2020) that was also identified in the present study was “usability heuristics.” This category refers to aspects related to the intrinsic properties of digital intervention programs that favor the development of users’ autonomy and their commitment to the therapeutic process. The five subcategories (“accessibility,” “interactivity,” “ease of use,” “aesthetic appeal,” and “self-directed”) that were described in the original model were also observed in our study, as explained below.

“Accessibility” appeared to refer to ease of access to the online intervention in accordance with participants’ needs and preferences, which facilitated their involvement in the therapeutic process. Within this subcategory, a new element was detected that had not been identified in the original model. This was a reference to the specific duration of the program and to the time in which patients continue to have access to the materials after completing the intervention. Depending on this time, participants would have more opportunities to understand and apply what was learned during the intervention, as illustrated by the following point:

*“Doing it at any time of the day has many advantages. I usually did it in the morning, which is when I’m most awake. That’s a very big advantage, and another advantage it also has is that you can review it, you can see it again. That’s also very important because there are some videos where I didn’t manage to capture everything the first time, what with my attention problem, and so now I’ve seen them more than once, you know. I know it’s a bonus.”* [Woman, 52 years old, self-guided application group, BDI score: 30 (12-month follow up)].

*“You really have to do this over and over; you really have to cram… because, you know, you’re doing it… and then you start to forget. I mean, at least for me… I’m doing it okay … and then it’s like ‘Wow! Anybody can do this!’ But later, if you’re feeling a bit down, for whatever reason, whatever, well then you actually have to … I think that in those cases, you’d need maybe a whole year, at least it should last longer, or you should have the chance to repeat it, I don't know! Or maybe keep it there a little longer, like saved*, *you know?”* [Man, 47 years old, low-intensity support group, BDI score: 14 (12-month follow up)].

In our study, the “interactivity” theme was defined as the ability of the program to react and produce comments and reflections from the information provided by users. In this respect, participants indicated that the digital intervention program was insufficient; they reported being left in doubts about theoretical aspects of the intervention, or with concerns that they could not express:

*“While I was doing the program, there would be times when I also needed an answer right away, you know. Or depending on how I felt, or how I saw certain things, mmm… Of course, the computer doesn’t answer your questions. So, you continue with the program, and you see that everything is very good and very clear. Until things come up that make you say, ‘What about this?’ Well, you leave them as they are because you can’t comment on them. So, contact is sometimes a good thing, don’t you think?"* [Woman, 46 years old, low-intensity support group, BDI score: 0 (12-month follow up)].

The “ease of use” subcategory was defined in the present study as having access to an intuitive digital interface. Participants suggested that the greater the feeling of control they are led to feel they have, the greater their motivation for completing the tasks of the program. Our data revealed a nuance to the theme that did not appear in the original model with regard to the importance of adapting the language to facilitate understanding of the information included in the program:

*“No difficulties; it was so easy to use. At least it was easy for me. Because, otherwise, it’s fine, very easy to do. It’s not complicated, well, maybe for someone who isn’t very familiar with computers. But I didn’t find it complicated at all"* [Woman, 55 years old, low-intensity support group, BDI score: 5 (12-month follow up)].

The specific nuance that was added to the model was:

*“Yes. It isn’t complicated. They don’t use weird words that you can’t understand. That’s what I was worried about at the beginning. I thought: ‘Huh, seeing that the stuff here is just like what doctors say, I’ll just answer the same as always: I don't know!’ But no, no, it’s okay… you can understand it perfectly.”* [Man, 55 years old, low-intensity support group, BDI score: 5 (12-month follow up)].

The “aesthetic appeal” subcategory referred to how attractive the digital psychotherapy program appeared to patients, to the extent that it influenced how useful they perceived the intervention to be:

*“In other words, it would have been a mistake to just have pages and pages to read; yes, I would have considered that to be a mistake. But the way they had it set up, I think they get to … get to the key issue of every point, and, I tell you, the accompanying videos and the like make it very enjoyable… and very easy, so you don’t mind repeating it, and watching it and going over it several times.”* [Woman, 44 years old, low-intensity support group, drop-out due to worsening symptoms, BDI score not available)].

The final subcategory, “self-directed,” was described as enabling in terms of developing a feeling of responsibility toward the therapeutic process, as well as a level of independence that empowers patients to engage with their own process. While self-directed treatment was perceived positively, there was an acknowledgement that strong willpower was required for any benefit to be possible:

*“The good thing about it is that you have to look at it yourself, and I also believe that where these things are concerned, you have to take responsibility for yourself. I think you have to give it your… a great deal of willpower"* [Woman, 46 years old, low-intensity support group, BDI score: 2 (12-month follow up)].

*“Your will to succeed gets you through it. When it comes to getting things done, it’s because you want to be cured or to improve.”* [Man, 47 years old, low-intensity support group, BDI score: 14 (12 months follow up)].

## 4. Discussion

This study is the first of its kind conducted in the PC setting in Spain that delves into the construct of the WA using an i-CBT intervention with patients allocated to receive low-intensity support or self-guided application for the treatment of mild to moderate depression. In line with Bordin’s theory (Bordin, 1979), and the framework developed by Doukani et al. for b-CBT (Doukani et al., 2020), our results confirm the relevance of “bond,” “goals” and “task” as essential elements for the establishment of the WA. This suggests a theoretical continuity from the conventional face-to-face therapeutic approach to new interventions that include a lower-intensity digital delivery applied through i-CBT. In addition, our findings endorse “usability heuristics” as an important and specific aspect of the WA in online interventions, as proposed by Doukani et al. (2020). However, they also expand the framework to include new nuanced accounts and subthemes that may be pertinent to the clinical population in Spain.

The “bond” category emerged as one of the main factors for establishing the WA, in comparison with previous models (Bordin, 1979; Doukani et al., 2020). Despite the general positive opinions of participants regarding the i-CBT program as a useful and even necessary tool, sole interaction with the computerized program was considered cold and unidirectional. Patients showed a certain preference for also having access to a professional “to count on” during the therapeutic process. This phenomenon appears to be associated with the emotional connection necessary to establish an adequate bond between the patient and the therapist and the program (Dunkle and Friedlander, 1996; Barazzone et al., 2016). In this regard, the provision of a space to feel heard and understood during the intervention, as proposed by the classic Bordin model, and also the model proposed by Doukani et al. for b-CBT (Bordin, 1979; Doukani et al., 2020), could be a central element. While our data confirmed the importance of “feeling understood,” as well as “genuineness” and “partnership,” compared with the model for b-CBT by Doukani et al. (2020) we found a new subtheme, need for “anonymity,” to be an important subcategory. Previous research has often described anonymity as a great advantage of Internet-delivered interventions for depression from the patient perspective (Wallin et al., 2016; Schröder et al., 2017). However, this need for anonymity appears to be associated with the feelings of shame and confusion that mental illness still aroused in the interviewed patients. It was interpreted as a general but substantive concern of patients regarding prejudice against and stigmatization of mental illness and the use of mental health services when receiving psychological treatment, perhaps as a consequence of the stigma that remains present in the Spanish society (Crespo et al., 2008; Pérez-Garín et al., 2015). A possible reason that would explain the absence of this subcategory in the United Kingdom context is the implementation of national strategies targeting the general population to reduce stigma and discrimination against people with mental disorders (Henderson and Thornicroft, 2011; Clement et al., 2013). Although the awareness and acceptance seems to be improving in the Spanish context, more needs to be done to address social stigma and discrimination at the population level (Rubio-Valera et al., 2016). Another explanation could be that the study by Doukani et al. allocated patients to either b-CBT or face-to-face treatment, whereas patients in the present study never saw the therapist; there was no physical contact with therapist in the low-intensity support group, and only email or phone interactions were used. Therefore, purely Internet-delivered approaches might somehow awaken a need for greater confidentiality (Dambi et al., 2023), and perhaps different delivery modes may invite different reactions in this regard.

“Goals” appeared to be an independent component, in line with Bordin’s model (Bordin, 1979). However, the nature of this component differed in that it seemed to be directly related to specific aspects of “tasks,” in the sense of “guidance,” since the interviewees indicated a greater need to have a person to help set the specific goals to achieve, rather than a need to delve into the reasons why the patient needs therapy. The same phenomenon was found in the WA model for b-CBT for depression developed by Doukani et al. (2020) as well as by the Working Alliance Inventory evaluation tool (Gómez Penedo et al., 2019). The structure of this measure brings together those aspects related to tasks and goals in the same factor. This may suggest that goal-setting dynamics involve different processes in the patient/therapist-program relationship, depending on whether or not it is a face-to-face relationship. In general, psychologists give a lot of importance to setting goals, which is a complicated task for patients to do alone. The results of the present study indicated that goal-setting might be one of the most important modules in a computerized program, since it allows a patient’s current situation to be determined, in addition to the changes the patient wishes to make. It is also one of the main sources of motivation for patients to commit to the intervention and engage with the program materials. However, as far as the computer psychotherapy program is concerned, participants stated that the lack of support with this part of the process can be a hindrance and may lead them to abandon the intervention.

The “task” category was also identified as one of the main elements necessary for the establishment of the WA. Furthermore, as observed in the United Kingdom study, the central task category was subdivided into two complementary subcategories (Doukani et al., 2020). On the one hand, task was directly related to the achievement of change (“activity-based task,” with its different aspects of “personalized,” “useful,” and “complementary”); and, on the other hand, task was specifically focused on the development of the WA (“responsive support,” with its corresponding characteristics of “accountability,” “guidance” and “expression of feelings”). Some elements of the task component of the WA model for b-CBT observed by Doukani et al. (2020) which have also been endorsed in the present study, were implicitly considered in Bordin’s original proposal (Bordin, 1979; e.g., “complementary tasks”). However, in the framework derived from b-CBT, these subthemes were explicitly presented and are therefore important entities in themselves (e.g., “accountability”). This phenomenon may be a consequence of the integration of technology and psychotherapy, which has given rise to new conditions that are to be considered for the development of the WA in online interventions (Mohr et al., 2011). Interestingly, [lack of] “guidance” was identified as a leading cause that explained why participants dropped out of the program. Our findings suggest that paying attention to this element in the design of Internet-delivered psychotherapy programs could help to develop and maintain the WA, and thus prevent the high dropout rate currently found in this type of psychological interventions (Andersson and Cuijpers, 2009; Saddichha et al., 2014; Montero-Marín et al., 2015).

Finally, a last category, called “usability heuristics,” which is also part of the WA model for b-CBT observed by Doukani et al. (2020) was endorsed by our findings. Similar to the task category, this was made up of the same type of subthemes of the original model, thus being validated in the present study. Two significant details that were observed in the present study regarding this category need to be specified. First, in terms of “accessibility,” patients expressed that they would prefer a longer duration for the intervention and to have access to the materials once they had completed the entire program, which would increase their opportunities to understand and apply what was learned. Second, in terms of “ease of use,” the program was valued positively, and participants highlighted the importance of using lay language at the nonexpert reader level to facilitate understanding. In summary, the “usability heuristics” category emerged as a new element of special importance with regard to Bordin’s original WA model (Bordin, 1979), since it encompasses characteristics of the digital program (e.g., “interactivity”) that directly influence the usability experience, which can therefore favor or hinder both the development of the WA and the participant’s involvement with and commitment to the entire therapeutic process.

The present study has several strengths. First, our study builds on Bordin’s WA model, which is widely accepted in the scientific and clinical community, as well as the recently development for b-CBT proposed by Bordin (1979) and Doukani et al. (2020). Second, our results are derived from the experiences provided by people who participated in an RCT to assess the efficacy of an Internet-delivered psychological intervention program for the treatment of depression. This enabled the study to engage in systematic data collection regarding the needs experienced and the interests of people who have suffered from the specific mental health problem. Finally, this study included both individuals who received low-intensity support from a professional and others who only had access to self-guided psychological intervention, so that its findings are supported by taking both modes of delivery into account. In this regard, we observed that some of the WA domains, such as “guidance,” might be responsible for creating different experiences between these two modes of delivery, which could affect the adherence to the program. Nevertheless, once the extended WA framework has been established for b-CBT/i-CBT, future studies should investigate how the different modes of delivery might potentially vary in terms of therapeutic experiences, and which specific WA domains might be responsible for those differences. There are other limitations that are important to note. In terms of representativeness, first, our study did not include the perspective of the clinical professionals, which limits the scope of the theoretical model and its possibilities to promote actions aimed at change and improvement. Second, our study largely comprised a relatively small number of middle-aged women living in urban areas, limiting the points of view of the population seeking and needing psychological treatment for depression in PC settings in Spain, and therefore restricting the replicability of the study results. Third, people who had little affinity with the use of specific technologies were excluded from the study, although this did not necessarily imply that they had no interest in the study and could benefit from this type of intervention. Future research will shed light on the extent to which the voices included in the present work have effectively crystallized into a valuable interpretation that coherently represents the WA experiences through Internet-delivered psychotherapy for depression in Spanish PC settings. Finally, the qualitative data analysis carried out avoided the use of superficial level themes (e.g., specific technological design). Instead, a latent thematic analysis was used to delve into the underlying psychological processes (Braun and Clarke, 2006).

The WA is a concept that has traditionally aroused the interest of both psychotherapists and researchers in the field of clinical psychology, given its relationship with symptom improvement and its predictive role, regardless of the type of intervention applied (Karver et al., 2006; Flückiger et al., 2012). Interestingly, the relationship between the WA and the improvement in symptoms of different psychological disorders has also been confirmed in studies where intervention has been delivered *via* the Internet, maintaining the association regardless of the specific symptoms, frequency, or mode of contact with the therapist (Kaiser et al., 2021).

In conclusion, the information derived from the present study is of great importance for clinical practice due to the new challenges posed by the integration of ICTs into the clinical field, specifically, in the treatment of psychological disorders, such as depression. Strengthening the development and maintenance of the WA using Internet-delivered programs could lead to a better experience of the therapeutic process and, consequently, improve the clinical impact of the treatment. Measures to enhance the WA using Internet-delivered programs should target the conventional domains of “goals,” “tasks,” and “bonds” as they are experienced in online psychotherapy, but they also need to specifically consider the “usability heuristics” domain by promoting active engagement, self-discovery, and autonomous problem-solving. Nevertheless, further efforts are required to develop a comprehensive understanding of the role of the WA in Internet-delivered psychological interventions. In this regard, quantitative research through the development of questionnaires to assess the WA in b-CBT/i-CBT is required to verify possible relationships between different online intervention deliveries and healthcare contexts, WA domains, and their impacts on the clinical improvement of symptoms.

## Data availability statement

The raw data supporting the conclusions of this article will be made available by the authors, without undue reservation.

## Ethics statement

The studies involving human participants were reviewed and approved by Research Ethics Committee of the regional health authority of the Autonomous Community of Aragon. The patients/participants provided their written informed consent to participate in this study.

## Author contributions

AB-S: formal analysis, data curation, and writing original draft. JG-C: conceptualization, methodology, investigation, resources, writing—review and editing, supervision, and project administration. RA and AD: writing—review and editing. MG, AG-P, and FM: conceptualization, investigation, resources, and supervision. JM-M: conceptualization, methodology, formal analysis, data curation, and writing—review and editing. All authors contributed to the article and approved the submitted version.

## Funding

This research has received funding from Government of Aragon (DGA) group (B17_20R) and the Network for Prevention and Health Promotion in Primary Care (RD16/0007/0005) grant from the Instituto de Salud Carlos III of the Spanish Ministry of Economy and Competitiveness, cofinanced with European Union ERDF funds. JM-M has a Miguel Servet contract from the Instituto de Salud Carlos III (ISCIII; CP21/00080). This study was supported by Institute for Health Research Aragon (IIS Aragón) and Chair of Contemplative Sciences and Master of Mindfulness of the University of Zaragoza, Spain. The funders did not have any influence on the results.

## Conflict of interest

The authors declare that the research was conducted in the absence of any commercial or financial relationships that could be construed as a potential conflict of interest.

## Publisher’s note

All claims expressed in this article are solely those of the authors and do not necessarily represent those of their affiliated organizations, or those of the publisher, the editors and the reviewers. Any product that may be evaluated in this article, or claim that may be made by its manufacturer, is not guaranteed or endorsed by the publisher.
